# Supplementary material for: COZOID: contact zone identifier for visual analysis of protein-protein interactions
Source: BMC Bioinformatics. 2018 Apr 6;19:125. doi: 10.1186/s12859-018-2113-6 (PMC5889581; doi:10.1186/s12859-018-2113-6)
Supplement: Supplementary file 2 — Software build. Executable binary file of the software tool. (ZIP 118,784 kb) [file 12859_2018_2113_MOESM2_ESM.zip › cozoid/java/docs/beansbinding-1.2.1-doc.zip!/index-files/index-5.html]

E-Index (Beans Binding)


---


|  |  |  |
| --- | --- | --- |
| **Overview** \| Package \| Class \| Use \| **Tree** \| **Deprecated** \| **Index** \| **Help** | |  |
| **PREV LETTER**   **NEXT LETTER** | **FRAMES**    **NO FRAMES**     **All Classes** |  |


A B C D E F G H I J K L M N O P Q R S T U V W 

---


## **E**

**ELArithmetic** - Class in org.jdesktop.el.impl.lang: A helper class of Arithmetic defined by the EL Specification **ELArithmetic()** - Constructor for class org.jdesktop.el.impl.lang.ELArithmetic: **ELArithmetic.BigDecimalDelegate** - Class in org.jdesktop.el.impl.lang: **ELArithmetic.BigDecimalDelegate()** - Constructor for class org.jdesktop.el.impl.lang.ELArithmetic.BigDecimalDelegate: **ELArithmetic.BigIntegerDelegate** - Class in org.jdesktop.el.impl.lang: **ELArithmetic.BigIntegerDelegate()** - Constructor for class org.jdesktop.el.impl.lang.ELArithmetic.BigIntegerDelegate: **ELArithmetic.DoubleDelegate** - Class in org.jdesktop.el.impl.lang: **ELArithmetic.DoubleDelegate()** - Constructor for class org.jdesktop.el.impl.lang.ELArithmetic.DoubleDelegate: **ELArithmetic.LongDelegate** - Class in org.jdesktop.el.impl.lang: **ELArithmetic.LongDelegate()** - Constructor for class org.jdesktop.el.impl.lang.ELArithmetic.LongDelegate: **ELContext** - Class in org.jdesktop.el: Context information for expression evaluation. **ELContext()** - Constructor for class org.jdesktop.el.ELContext: **ELContextEvent** - Class in org.jdesktop.el: An event which indicates that an `ELContext` has been created. **ELContextEvent(ELContext)** - Constructor for class org.jdesktop.el.ELContextEvent: Constructs an ELContextEvent object to indicate that an `ELContext` has been created. **ELContextListener** - Interface in org.jdesktop.el: The listener interface for receiving notification when an `ELContext` is created. **ELException** - Exception in org.jdesktop.el: Represents any of the exception conditions that can arise during expression evaluation. **ELException()** - Constructor for exception org.jdesktop.el.ELException: Creates an `ELException` with no detail message. **ELException(String)** - Constructor for exception org.jdesktop.el.ELException: Creates an `ELException` with the provided detail message. **ELException(Throwable)** - Constructor for exception org.jdesktop.el.ELException: Creates an `ELException` with the given cause. **ELException(String, Throwable)** - Constructor for exception org.jdesktop.el.ELException: Creates an ELException with the given detail message and root cause. **ELParser** - Class in org.jdesktop.el.impl.parser: **ELParser(InputStream)** - Constructor for class org.jdesktop.el.impl.parser.ELParser: **ELParser(Reader)** - Constructor for class org.jdesktop.el.impl.parser.ELParser: **ELParser(ELParserTokenManager)** - Constructor for class org.jdesktop.el.impl.parser.ELParser: **ELParserConstants** - Interface in org.jdesktop.el.impl.parser: **ELParserTokenManager** - Class in org.jdesktop.el.impl.parser: **ELParserTokenManager(SimpleCharStream)** - Constructor for class org.jdesktop.el.impl.parser.ELParserTokenManager: **ELParserTokenManager(SimpleCharStream, int)** - Constructor for class org.jdesktop.el.impl.parser.ELParserTokenManager: **ELParserTreeConstants** - Interface in org.jdesktop.el.impl.parser: **ELProperty**<S,V> - Class in org.jdesktop.beansbinding: An implementation of `Property` that allows Java Beans properties of source objects to be addressed using a simple dot-separated path syntax within an EL expression. **ELResolver** - Class in org.jdesktop.el: Enables customization of variable and property resolution behavior for EL expression evaluation. **ELResolver()** - Constructor for class org.jdesktop.el.ELResolver: **ELSupport** - Class in org.jdesktop.el.impl.lang: A helper class that implements the EL Specification **ELSupport()** - Constructor for class org.jdesktop.el.impl.lang.ELSupport: **EMPTY** - Static variable in interface org.jdesktop.el.impl.parser.ELParserConstants: **EMPTY\_STRING** - Static variable in class org.jdesktop.el.impl.util.ReflectionUtil: **enable\_tracing()** - Method in class org.jdesktop.el.impl.parser.ELParser: **END\_EXPRESSION** - Static variable in interface org.jdesktop.el.impl.parser.ELParserConstants: **endColumn** - Variable in class org.jdesktop.el.impl.parser.Token: beginLine and beginColumn describe the position of the first character of this token; endLine and endColumn describe the position of the last character of this token. **endLine** - Variable in class org.jdesktop.el.impl.parser.Token: beginLine and beginColumn describe the position of the first character of this token; endLine and endColumn describe the position of the last character of this token. **EOF** - Static variable in interface org.jdesktop.el.impl.parser.ELParserConstants: **eol** - Variable in exception org.jdesktop.el.impl.parser.ParseException: The end of line string for this machine. **EQ0** - Static variable in interface org.jdesktop.el.impl.parser.ELParserConstants: **EQ1** - Static variable in interface org.jdesktop.el.impl.parser.ELParserConstants: **Equality()** - Method in class org.jdesktop.el.impl.parser.ELParser: **equals(Object)** - Method in class org.jdesktop.el.Expression: Determines whether the specified object is equal to this `Expression`. **equals(Object)** - Method in class org.jdesktop.el.Expression.ResolvedProperty: **equals(Object, Object)** - Static method in class org.jdesktop.el.impl.lang.ELSupport: **equals(Object)** - Method in class org.jdesktop.el.impl.lang.FunctionMapperImpl.Function: **equals(Object)** - Method in class org.jdesktop.el.impl.MethodExpressionImpl: Determines whether the specified object is equal to this `Expression`. **equals(Object)** - Method in class org.jdesktop.el.impl.MethodExpressionLiteral: **equals(Object)** - Method in class org.jdesktop.el.impl.ValueExpressionImpl: **equals(Object)** - Method in class org.jdesktop.el.impl.ValueExpressionLiteral: **equals(ValueExpressionLiteral)** - Method in class org.jdesktop.el.impl.ValueExpressionLiteral: **EvaluationContext** - Class in org.jdesktop.el.impl.lang: **EvaluationContext(ELContext, FunctionMapper, VariableMapper, Expression)** - Constructor for class org.jdesktop.el.impl.lang.EvaluationContext: **EvaluationContext(ELContext, FunctionMapper, VariableMapper, Expression, boolean)** - Constructor for class org.jdesktop.el.impl.lang.EvaluationContext: **ExpandBuff(boolean)** - Method in class org.jdesktop.el.impl.parser.SimpleCharStream: **expectedTokenSequences** - Variable in exception org.jdesktop.el.impl.parser.ParseException: Each entry in this array is an array of integers. **EXPONENT** - Static variable in interface org.jdesktop.el.impl.parser.ELParserConstants: **Expression** - Class in org.jdesktop.el: Base class for the expression subclasses `ValueExpression` and `MethodExpression`, implementing characterstics common to both. **Expression()** - Constructor for class org.jdesktop.el.Expression: **Expression()** - Method in class org.jdesktop.el.impl.parser.ELParser: **Expression.ResolvedProperty** - Class in org.jdesktop.el: **Expression.ResolvedProperty(Object, Object)** - Constructor for class org.jdesktop.el.Expression.ResolvedProperty: **Expression.Result** - Class in org.jdesktop.el: **Expression.Result(Expression.Result.Type, Object, List<Expression.ResolvedProperty>)** - Constructor for class org.jdesktop.el.Expression.Result: **Expression.Result.Type** - Enum in org.jdesktop.el: **ExpressionBuilder** - Class in org.jdesktop.el.impl.lang: **ExpressionBuilder(String, ELContext)** - Constructor for class org.jdesktop.el.impl.lang.ExpressionBuilder: **ExpressionFactory** - Class in org.jdesktop.el: Parses a `String` into a `ValueExpression` or `MethodExpression` instance for later evaluation. **ExpressionFactory()** - Constructor for class org.jdesktop.el.ExpressionFactory: **ExpressionFactoryImpl** - Class in org.jdesktop.el.impl: **ExpressionFactoryImpl()** - Constructor for class org.jdesktop.el.impl.ExpressionFactoryImpl

---


|  |  |  |
| --- | --- | --- |
| **Overview** \| Package \| Class \| Use \| **Tree** \| **Deprecated** \| **Index** \| **Help** | |  |
| **PREV LETTER**   **NEXT LETTER** | **FRAMES**    **NO FRAMES**     **All Classes** |  |


A B C D E F G H I J K L M N O P Q R S T U V W 

---
